# Individual and community-level determinates of risky sexual behaviors among sexually active unmarried men: A multilevel analysis of 2016 Ethiopian Demographic and Health Survey

**DOI:** 10.1371/journal.pone.0270083

**Published:** 2022-07-21

**Authors:** Gedefaw Diress, Seteamlak Adane, Melese Linger, Abebe Merchaw, Belayneh Mengist

**Affiliations:** 1 Department of Public Health, College of Health Science, Debre Markos University, Debre Markos, Ethiopia; 2 School of Public Health, College of Health Science, Woldia University, Woldia, Ethiopia; 3 Department of Human Nutrition, College of Health Science, Debre Markos University, Debre Markos, Ethiopia; 4 School of Nursing, College of Health Science, Woldia University, Woldia, Ethiopia; Indiana University School of Medicine, UNITED STATES

## Abstract

**Background:**

In Ethiopia, HIV/AIDS continues to be a major public health problem mostly due to the high prevalence of risky sexual behaviors. However, research on risky sexual behavior and its determinants among unmarried men (never married, widowed, and divorced) who are highly vulnerable to risky sexual behavior was limited. Therefore, this study aimed to assess the magnitude of risky sexual behavior and its determinants among non-married men using a nationally representative sample.

**Methods:**

The analysis was done on 5680 sexually active unmarried men aged 15–59 years using data from the 2016 Ethiopia Demographic Health Survey (EDHS). The main outcome variable was risky sexual behavior which defined as having at least one of the following: multiple sexual partners; initiation of sex before the age of 18 years; inconsistent condom use in the last 12 months; alcohol consumption at last sex. Multivariable generalized linear mixed-effects regression was employed to identify variables associated with risky sexual behavior.

**Result:**

The overall magnitude of risky sexual behavior was 26.9% (95% CI; 25.7, 28.0). Currently employed (AOR = 2.49, 95% CI = 1.64–3.77), history of HIV testing (AOR = 2.51, 95% C = 1.95–3.23), drinking alcohol almost every day (AOR = 5.49, 95 CI = 2.73–11.02), and using Internet daily (AOR = 1.99, 95% CI = 1.06–3.74) increase the odds of risky sexual behavior. Whereas, primary education (AOR = 0.44, 95% CI = 0.32–0.61), secondary education level (AOR = 0.46, 95% CI = 0.29–0.72) and a high proportion of community-level media exposure (AOR = 0.42, 95% CI = 0.12–0.75) decrease the odds of risky sexual behavior.

**Conclusion:**

In general, a significant proportion of sexually active unmarried men in Ethiopia have practiced risky sexual behavior. An intervention should be designed which are against the factors found to increase the odds of risky sexual behavior to reduce the incidence of HIV and other sexually transmitted infections.

## Introduction

Risky sexual behavior is characterized by different harmful behaviors such as early sexual debut, multiple sexual partners, and unprotected sex which can result in unintended health outcomes like HIV/AIDS, unwanted pregnancies, and unsafe abortions [1]. In sub-Saharan Africa including Ethiopia, HIV/AIDS continues to be a major public health problem mostly due to the high prevalence of these risky sexual behaviors, particularly inconsistent condom use and having multiple sexual partners [2].

In Ethiopia, the trends in risky sexual behavior among the sexually active segment of the population are increasing alarmingly [3, 4]. A study conducted on male and female youth(15–24 year age group) in Addis Ababa (the capital city of Ethiopia) revealed that 43% of youth have reported having risky sexual behavior [5], 37% of youth started sexual intercourse before the age of 18 years, 47% of the youth did not use a condom during their sexual intercourse in the past 12 months and 42% of youth had more than one sexual partners in the past 12 months before the survey [5]. Similarly, a recent study done in northern parts of Ethiopia showed that 27.5% of youth have practiced risky sexual behavior. More than one-third of youths have started their first sexual intercourse before 18 birthdays. Among participants who had a history of sexual exposure, 25.5% of first sexual intercourse was unplanned and undecided or unconvinced. Even though the first sexual intercourse in a significant number of youth was unplanned, only 21.6% of them have used condoms during their first sexual intercourse [6]. In the 2016 Ethiopian national survey, 7% of men aged 15–49 years had sexual intercourse in the past 12 months with a person who was neither their wife nor lived with them, but nearly half (49%) of them reported not using a condom during the last sexual intercourse with such a partner [7]. Therefore, identifying the facilitators of risky sexual behavior among sexually active men is needed in Ethiopia. Besides, marital status-specific information on the magnitude of risky sexual behavior and its determinants is necessary to reduce unwanted and negative consequences of risky behaviors. Past studies reported mixed results on the association between marital status and levels of risky sexual behavior. Different studies conducted in different parts of the globe reported that being married increased the likelihood of risky sexual behavior [7–10]. In Ethiopia, similarly, the 2016 EDHS reported that men who are married are more likely to have more than one partner in the past 12 months than those who were never married (4% compared to 2%) [7].

On the contrary, few recent studies reported that unmarried people (never married, widowed, and divorced) are particularly vulnerable to risky sexual behavior compared to married, leading to a high HIV incidence rate among the unmarried groups of the population [11, 12]. A recent study done among American men revealed that men who were married reported the lowest levels of risk behavior as compared to unmarried ones [11]. The plausible explanation for this is marriage makes a stable sexual network between couples. People with more sexual partners may be at higher risk of contracting sexually transmitted infections including HIV/AIDS than those with reliable partners [13]. It is acceptable that single/divorced/separated persons have a wider sexual network, leading to more sexual partners, which in turn increases their risk of acquiring HIV/AIDS. Similarly, in Ethiopia, the unmarried population, particularly those cohabitating with one another without marriage, were targeted for HIV prevention because HIV incidence among this group is the highest in the country [7, 14]. However, in the country, researchers who have identified the factors affecting risky sexual behavior focused on university and high school students, as a unit of analysis; unmarried sexually active men in the general population tend to be ignored in most studies [15–17].

Sexual attitude and behavior can vary significantly between males and females. Past evidence suggests that there are significant gender differences in risky sexual behavior (e.g., multiple sex partners, inconsistent condom use, non-use of contraceptives) between males and females. Young men tend to become sexually active earlier and had more multiple sexual partners than young women [18]. Based on the 2016 Ethiopian Demographic Health Survey (EDHS) report, men initiate sexual intercourse 2.5 years before marriage. In contrast, among women, the median age at first intercourse is 0.5 years younger than the median age at first marriage [7]. Besides, because of differences with regards to physical excitement and drug consumption, men have a greater tendency to have sex with casual partners than women. In addition, in Ethiopia, social norms encourage young girls to refrain from having sex and avoid multiple sexual partners. Men’s risky sexual behavior contributes to the spread of HIV in the country [19]. Therefore, understanding the determinants of risky sexual behavior is essential to designing an appropriate intervention plan to reduce the prevalence of risky sexual behavior among the sexually active segment of the population, particularly unmarried men.

**Fig 1:**
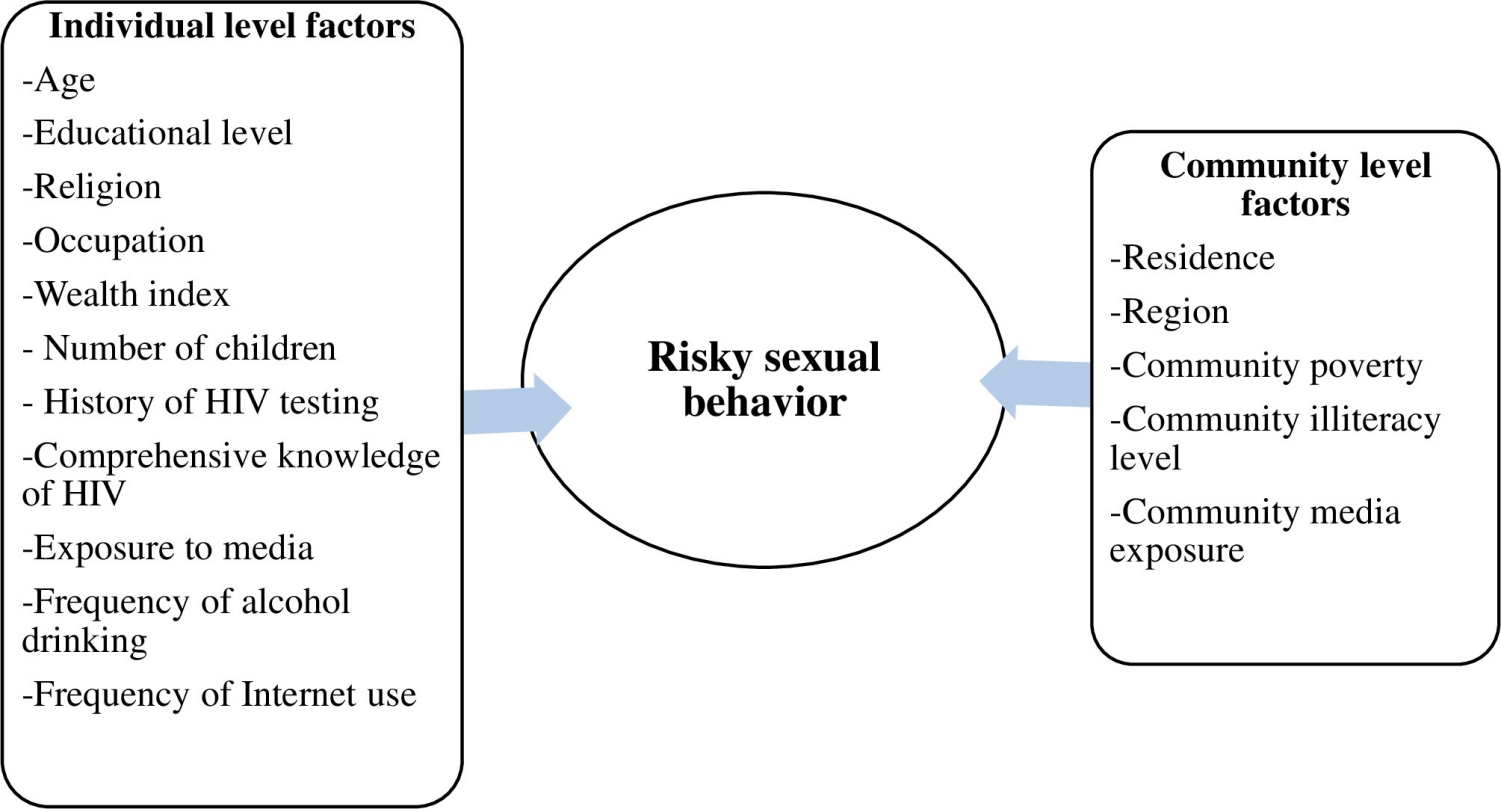
A conceptual framework for determinants of risky sexual behavior among unmarried men in Ethiopia.

Research on risky sexual behavior and its determinants (Fig 1) among never-married, divorced and widowed groups of men in Ethiopia were limited. Especially, the community-level determinants of risky sexual behavior remain poorly understood. Therefore, this study aimed to assess the magnitude of risky sexual behavior and its determinants both at the individual and community level among unmarried groups using a nationally representative sample.

## Materials and methods

This analysis used secondary data from the 2016 EDHS. A detailed explanation of the methodology of 2016 EDHS is found elsewhere [7]. Data were obtained from the DHS program website: https://www.dhsprogram.com. Our analysis sample consisted of unmarried sexually active men (individuals who are never in a union, divorced, widowed, and separated) aged 15–59 years. After excluding men who were married at the time of the survey, 5680 men were eligible for the final analysis.

### Study population and eligibility criteria

The source population was all sexually active unmarried men (aged 15–59 years) in Ethiopia. Eligibility criteria included being in the age group of 15–59 years and being unmarried during the survey. Sexually active unmarried men include men who are not currently married or in a consensual union (single, divorced, widowed, and separated) and who had sexual intercourse within the last 30 days.

### Study variables

#### Outcome variable

One binary outcome variable was created that is risky sexual behavior defined as having at least one of the following: multiple sexual partners; initiation of sex before the age of 18 years; inconsistent condom use in the last 12 months; alcohol consumption at last sex. In the analysis section, the outcome variable refers to this created binary outcome variable (risky sexual behavior (Yes or No)).

The 2016 EDHS asked the number of sex partners, excluding the spouse, in the last 12 months. Based on this, having multiple sexual partners was defined as having two or more sexual partners in the last 12 months. Similarly, the 2016 EDHS asked the age at which respondents first had sexual intercourse. The definition of early sexual initiation might vary according to the social and demographic context of the nation. However, the Universal Declaration of Human Rights proclaimed as an age below 18 years old is considered as a child, they couldn’t decide concerning marriage and/or consensual sexual relationship [20]. Similarly, in Ethiopia, the minimum age of marriage is still 18 years and sexual initiation before 18 years old, most often, is considered as a risky sexual behavior because of its adverse consequences. Therefore, in this study, we considered a sexual activity that began earlier than 18 years of age as early sexual initiation [21, 22]. The answer to having sexual intercourse before age 18 years was dichotomized, with 0 indicating no initiation of sex before 18 years of age and 1 indicating yes.

Inconsistent condom use was computed from the question: Was a condom used every time you had sexual intercourse with three most recent partners in the last 12 months? Inconsistent condom use was defined if the participants did not use a condom every time they had sexual intercourse with the three most recent partners in the last 12 months. Alcohol consumption at last sex with the most recent partner was based on the question “Had you been drinking any alcohol the last time you had sexual intercourse?” The response was dichotomized as yes or no.

#### Explanatory variables

Consistent with the research objective, two levels of explanatory variables were considered.

*Individual-level characteristics (Level 1)*. include age (15–24, ≥25), level of education (no education, primary, secondary and higher), religion (Orthodox, Protestant, Muslim, and others), number of children (none, 1–2, ≥ 3), occupation (working or not working), wealth index (poor, middle, and rich), media exposure (yes, no), comprehensive knowledge to HIV (yes, no), frequency of alcohol consumption in the last 12 months (almost every day, at least once a week, less than once a week, not at all/none in the last 12 months), frequency of internet use in the last month (not at all, less than once a week, at least once a week and almost every day), ever tested for HIV(yes/ no), ever heard about STI (yes/no).

*Community-level characteristics (Level 2)*. include residence (urban and rural), region, community poverty, community illiteracy rate, and community level of media exposure. Residence and region were used without manipulation. However, the aggregate community-level variables like community poverty, community illiteracy rate, and community level of media exposure were computed by aggregating individual-level characteristics at the cluster level.

Regarding occupation/employment status; ‘worked’ was defined as men who worked in the 12 months preceding the survey. This variable is included because it is a source of income for men.

Individual-level media exposure was classified based on response to how often respondents read a newspaper, listened to the radio, or watched television. Those who responded at least once a week to any of these sources were considered to have access to media.

The frequency of alcohol consumption was categorized based on response to “how often they drank alcohol in the last 12 months prior to the survey?” and four response categories were included: almost every day, at least once a week, less than once a week, and not at all or none in the last 12 months.

Comprehensive knowledge on HIV/AIDS was defined based on a widely used measure where each participant was asked whether or not he agreed or disagreed with the following five items. (1.) Consistent use of condoms during sexual intercourse can reduce the chance of getting HIV, (2.) having just one uninfected faithful partner can reduce the chance of getting HIV, (3.) a healthy-looking person can have HIV, (4.) HIV can be transmitted by mosquito bites and (5.) a person can become infected by sharing food with a person who has HIV. An additive summary score was created and which was then dichotomized to create a binary variable with 0 indicating at least one incorrect response and 1 indicating a correct response to five items (Having a comprehensive knowledge of HIV was defined if respondents responded to all five questions correctly).

Community poverty level, community illiteracy level and community media were dichotomized as high or low based on the distribution of the proportion values computed for each community after checking the distribution by using the histogram. If the aggregate variable was normally distributed mean value and if not, the normally distributed median value was used as a cut-off point for the categorization. In the current study, all these three community-level variables were dichotomized as high or low, based on the median value because all aggregated variable was not normally distributed. The median value for the proportion of community poverty level, illiteracy level and community media exposure was 30.7%, 23.5%, and 56.0% respectively.

Community poverty level was categorized as low if the proportion of men from the two lowest wealth quintiles (poorest and poor) in a given cluster was 0–30.7% and high if the proportion was above 30.7%. Community illiteracy level was categorized as low if the proportion of illiterate men in a given community was 0–23.5% and high if it was greater than 23.5%. Community media exposure was categorized as low if the proportion of men exposed to media in the community was 0–56.0% and categorized as high if the proportion was above 56.0%. All these three community-level variables (poverty level, illiteracy level, and media exposure) were dichotomized as high or low, based on the median value after checking their distribution (all aggregated variable was not normally distributed).

### Statistical analysis

The EDHS samples were not self-weighted due to the non-proportional allocation of the sample to different regions as well as urban and rural areas, and the possible differences in response rates. Thus, the data were weighted before doing any statistical analysis to restore the representativeness of the sample and get a reliable estimate and standard error. In this study, the weight variable was created by dividing the individual weight for men by 1000,000. A detailed explanation of the weighting procedure can be found in the DHS guide [23].

Participant characteristics were summarized using frequency and weighted percentage. Multi-collinearity between independent variables was cheeked before fitting the final regression model. When two independent variables were found highly correlated, one was dropped.

#### Model building

To choose the best-fitted model, four models containing variables of interest were fitted and compared them with deviance. The first was the null model (Model I) containing no exposure variables which was used to check the variability of risky sexual behavior among the communities. The second (Model II) is a multivariate model adjustment for individual-level variables which had a p-value of less than 0.2 in the bivariable model. Hence, all individual-level independent variables which were significant in model-II (i.e. multivariable model adjustment for individual-level variables) were considered as candidates for the final model. The third (Model III) multilevel models contain community-level variables which had a p-value of less than 0.2 in the bivariable model. All independent variables which were statistically significant in model-3 (community-level variables model adjustment) were included in the final model as potential candidates. In the fourth model (Model IV) both individual and community level variables were fitted simultaneously with the outcome variable. Some of the community-level variables like community illiterate level and community poverty level were computed from individual-level variables such as educational status and wealth index respectively. In the regression model, we used individual level and community level factors in parallel.

DHS data are correlated in nature, therefore, the community variation (random effect) was measured in the terms of Intra Community Correlation Coefficient (ICC), Proportional Change in Community-level variance (PCV), and median odds ratio (MOR).

ICC is the measure of the percentage variance explained by the community-level variables, while PCV measures the proportional change in the community-level variance between the empty model and the subsequent models. The MOR aims to translate the community level variance in the widely used odds ratio scale, which has a consistent and intuitive interpretation. The MOR is defined as the median value of the odds ratio between the area at the highest risk and the area at the lowest risk when randomly picking out two areas or clusters. Log-likelihood and Akaike’s Information Criterion (AIC) were used as model fit statistics.

### Ethics approval

The study was based on the analyses of existing survey datasets with all identifier information removed. The surveys were approved by the Ethiopia Health and Nutrition Research Institute Review Board, the National Research Ethics Review Committee at the Ministry of Science and Technology, and the Institutional Review Board of ICF International, and the Centers for Disease Control and Prevention. Informed verbal consent was obtained from all men, and recorded by the research team on the ethical consent form. The investigators have accessed the EDHS survey data from the DHS program website: https://www.dhsprogram.com and then the researchers have maintained the confidentiality of the data.

## Result

### Sociodemographic characteristics of study participants

In this study, we used weighted samples of 5680 unmarried sexually active men. The median age of the study participants was 20 years (IQR = 17–25). The majority (47.1%) of participants had completed primary education and about half (47.0%) of respondents were followers of orthodox religion. Regarding the wealth index, the majority (57.6%) of males were in the rich wealth index category. Most (52.8%) of respondents had no media exposure and about 59.5% of study subjects had no comprehensive knowledge of HIV. The majority (58.6%) of unmarried sexually active men had not ever been tested for HIV. About three-fourths (73.5%) of participants didn’t use the Internet at all last month prior to the survey. Regarding respondents’ residence, 61.6% of respondents were from rural residences (Table 1).

**Table 1:** Sociodemographic and community-level characteristics of unmarried men in Ethiopia, 2016.

Variables	Category	Frequency (weighted)	Percentage (weighted)
**Age**	15–24	4014	70.7
	> = 25	1666	29.3
**Educational level**	No formal education	720	12.7
	Primary education	2677	47.1
	Secondary education	1444	25.4
	Higher education	839	14.8
**Occupation**	Not working	1338	23.6
	Working	4342	76.4
**Religion**	Orthodox	2667	47.0
	Protestant	930	16.4
	Muslim	1979	35.0
	Others	104	1.6
**Wealth index**	Poor	1708	30.1
	Middle	699	12.3
	Rich	3273	57.6
**Media exposure**	No	3000	52.8
	Yes	2680	47.2
**Comprehensive HIV knowledge**	No	3380	59.5
	Yes	2300	40.5
**Ever heard of STI**	No	221	3.9
	Yes	5459	96.1
**Ever been tested for HIV**	No	3327	58.6
	Yes	2353	41.4
**Frequency of alcohol drinking in the last 12 months**	Not at all/none in the 12 months	3340	58.8
	Less than once a week	1132	19.9
	At least once a week	1094	19.3
	Almost every day	114	2.0
**Frequency of using internet last month**	Not at all	4173	73.5
	Less than once a week	491	8.6
	At least once a week	410	7.2
	All most every day	606	10.7
**Residence**	Urban	2183	38.4
	Rural	3497	61.6
**Community illiteracy level**	Low	3746	66.0
	High	1934	34.0
**Community poverty level**	Low	3226	56.8
	High	2454	43.2
**Community media exposure**	Low	3160	55.6
	High	2520	44.4
**Region**	Tigray	297	5.2
	Afar	133	2.3
	Amhara	1246	21.9
	Oromia	1382	24.3
	Somali	725	12.8
	Benishangul	196	3.5
	SNNPR	1153	20.3
	Gambela	241	4.2
	Harari	98	1.7
	Addis Ababa	142	2.5
	Dire Dewa	67	1.2

### Magnitude of risky sexual behavior among unmarried men

The overall magnitude of risky sexual behavior was 26.9% (95% CI; 25.7, 28.0). In this study, 25.5% of the unmarried men had a sexual debut before the age of 18 years and about 3.4% had multiple sexual partners. In this study, 15.1% of the respondents inconsistently used condoms in their most recent sexual intercourse.

The highest prevalence of risky sexual behavior was observed in Addis Ababa (47.5%) followed by the Afar region (45.7%).

### Random effect and model comparison

As indicated in Table 2, the ICC in the null model was 24.5, which means about 25% of the variations of risky sexual behavior among unmarried men were attributable to the difference at cluster level factors. The higher MOR value (3.31) in the null model also revealed that risky sexual behavior among unmarried men was different between clusters. Furthermore, the higher PCV value (0.28) in the final model indicates that about 28% of the variation of risky sexual behavior among unmarried men was attributable to both the individual level and community level factors (Table 2).

**Table 2:** Random effect and model comparison for factors associated with risky sexual behavior among sexually active unmarried men in Ethiopia, 2016.

**Random effect**	**Model I**	**Model II**	**Model III**	**Model IV**
ICC (%) (95% CI)	24.5(20.3–29.2)	20.8(16.4–27.4)	17.1(13.5–21.6)	19.0(14.0–25.2)
PCV (%)	Reference	19.2	36.4	28.0
MOR	3.31	2.95	2.61	2.77
Community variance (SE)	1.065(0.1304)*	0.865(0.1527)	0.681(0.0992)	0.769(0.1435)
**Model fitness**	**Model I**	**Model II**	**Model III**	**Model IV**
Log likelihood	-3190.7166	-2274.694	-3024.1453	-2215.8041
AIC	5835.501	4312.375	5718.364	4285.273
BIC	5848.79	4431.98	5824.68	4511.193

Abbreviation: ICC-intra-cluster correlation; PCV-proportional change in variance; MOR-median odds ratio; AIC; Akaike information criteria; SE-standard error

*Statistical significance at 5% level p<0.05.

### Factors associated with risky sexual behavior

In this study, the results of the empty model (Model I) showed that there was statistically significant variability in the odds of having risky sexual behavior (ICC = 25%) which is the total variance in the risky sexual behavior was attributed to differences between clusters.

In Model II, only individual-level variables were added. The results showed that age ≥25 years (AOR = 10.8, 95% CI = 7.77–15.1), currently working (AOR = 2.55, 95% CI = 1.68–3.89), tested for HIV (AOR = 2.64, 95% CI = 2.05–3.40), alcohol drinking almost every day (AOR = 5.49, 95% CI = 2.73–11.02) and use the Internet almost every day (AOR = 2.26, 95% CI = 1.22–4.20) increase the odds of having risky sexual behavior. However, primary level of education (AOR = 0.47 95% CI = 0.34–0.65), secondary level of education (AOR = 0.51, 95% CI = 0.33–0.78), being Protestant (AOR = 0.62, 95% CI = 0.43–0.92) and being Muslim (AOR = 0.56, 95% CI = 0.38–0.84) decrease the odds of risky sexual behavior.

In Model III, community-level variables were included. The results showed that rural residence (AOR = 0.62, 95% CI = 0.41–0.94), high level of poverty in the community (AOR = 0.58, 95% CI = 0.40–0.82), and region were significantly associated with risky sexual behavior.

In the final model (Model IV), both individual and community-level factors were included simultaneously. The result revealed that level of education, working status, religion, wealth index, history of HIV testing, frequency of internet use, community level media exposure, and region were significantly associated with risky sexual behavior.

After controlling for other individual and community level factors, sexually active men who had primary education were 56% (AOR = 0.44, 95% CI = 0.32–0.61) and men who had secondary education were 54% (AOR = 0.46, 95% CI = 0.29–0.72) reduction in the odds of having risky sexual behavior as compared to men who had no any formal education. Regarding working status, men who are currently working were 2.5 times (AOR = 2.49, 95% CI = 1.64–3.77) more likely to have high odds of risky sexual behavior as compared to those not working. After holding other factors constant, men from middle wealth index households had 37% lower (AOR = 0.63, 95% CI = 0.41–0.96) odds of risky sexual behavior as compared to men from poor households. Men who had ever been tested for HIV were 2.5 times (AOR = 2.51, 95% C = 1.95–3.23) more odds to have risky sexual behavior as compared to their counterparts. Men who drank alcohol almost every day in the12 months prior to the survey were 5.5 times (AOR = 5.49, 95 CI = 2.73–11.02) more likely to have risky sexual behavior as compared to nondrinkers. Similarly, men who used Internet almost every day in the last month prior to the survey were 2 times (AOR = 1.99, 95% CI = 1.06–3.74) more likely to have risky sexual behavior as compared to non-users.

Men residing in communities with a high proportion of media exposure had a 58% (AOR = 0.42, 95% CI = 0.12–0.75) reduction in the odds of having risky sexual behavior as compared to men residing in communities with a low proportion of media exposure. Men living in Tigray, Amhara, Oromia, Somalia, SNNP, and Harari regional states had a lower odds of having risky sexual behavior as compared to men who lived in Addis Ababa(capital city of the country) (Table 3).

**Table 3:** A multivariable multilevel analysis of factors associated with risky sexual behavior among men in Ethiopia, 2016.

Variables	Category	Model I	Model II AOR (95% CI)	Model III AOR (95% CI)	Model IV AOR (95% CI)
**Age**	15–19		Ref.		Ref.
	> = 25		2.8(1.77–3.71)***		1.70(0.83–1.87)
**Educational level**	No formal education		Ref.		Ref.
	Primary education		0.47(0.34–0.65)***		0.44(0.32–0.61)***
	Secondary education		0.51(0.33–0.78)**		0.46(0.29–0.72)**
	Higher education		0.75(0.40–1.44)		0.69(0.36–1.32)
**Occupation**	Not working		Ref.		Ref.
	Working		2.55(1.68–3.89)***		2.49(1.64–3.77)***
**Religion**	Orthodox		Ref.		Ref.
	Protestant		0.62(0.43–0.92)*		0.70(0.45–1.08)
	Muslim		1.21(0.88–1.84)		1.55(1.15–2.87)*
	Others		0.24(0.08–0.70)**		0.28(0.09–0.84)*
**Wealth index**	Poor		Ref.		Ref.
	Middle		0.66(0.44–1.01)		0.63(0.41–0.96)*
	Rich		0.87(0.59–1.26)		0.74(0.47–1.16)
**Media exposure**	No		Ref.		Ref.
	Yes		1.23(0.91–1.66)		1.12(0.82–1.54)
**Comprehensive HIV knowledge**	No		Ref.		Ref.
	Yes		1.08(0.85–1.37)		1.01(0.80–1.30)
**Ever heard of STI**	No		Ref.		Ref.
	Yes		1.23(0.67–2.10)		2.78(0.55–14.0)
**Ever been tested for HIV**	No		Ref.		Ref.
	Yes		2.64(2.05–3.40)***		2.51(1.95–3.23)***
**Frequency of alcohol drinking in the last 12 months**	None^a^				Ref.
	Less than once a week		3.74(2.78–5.03)***		2.37(1.48–3.80)**
	At least once a week		1.78(1.32–2.40)**		1.38(0.86–2.23)
	Almost every day		5.49(2.73–11.02)***		3.77(1.55–9.20)***
**Frequency of using internet last month**	Not at all		Ref.		Ref.
	Less than once a week		1.51(0.81–2.84)		1.40(0.75–2.61)
	At least once a week		1.63(0.93–2.88)		1.44(0.80–2.56)
	All most every day		2.26(1.22–4.20)**		1.99(1.06–3.74)*
**Residence**	Urban			Ref.	Ref.
	Rural			0.62(0.41–0.94)*	1.19(0.71–1.98)
**Community illiteracy level**	Low			Ref.	Ref.
	High			1.12(0.79–1.59)	0.98(0.65–1.45)
**Community poverty level**	Low			Ref.	Ref.
	High			0.58(0.40–0.82)**	0.62(0.40–1.98)
**Community media exposure**	Low			Ref.	Ref.
	High			1.16(0.82–1.62)	0.42(0.12–0.75)**
**Region**	Tigray			0.45(0.31–0.66)***	0.56(0.35–0.90)*
	Afar			0.66(0.39–1.10)	1.14(0.57–2.30)
	Amhara			0.28(0.18–0.44)***	0.34(0.20–0.57)***
	Oromia			0.43(0.21–0.83)***	0.57(0.33–0.98)*
	Somali			0.40(0.26–0.60)***	0.36(0.17–0.75)**
	Benishangul			0.48(0.31–0.74)**	0.65(0.39–1.09)
	SNNPR			0.26(0.13–0.35)***	0.30(0.16–0.54)***
	Gambela			0.50(0.33–0.74)**	0.80(0.48–1.31)
	Harari			0.32(0.21–0.48)***	0.46(0.27–0.78)**
	Addis Ababa			Ref.	Ref.
	Dire Dewa			0.46(0.32–0.64)***	0.70(0.48–1.03)

**Note**: AOR-Adjusted Odds Ratio, CI- Confidence Interval, SNNPR-southern nation nationalities and people; STI-sexually transmitted infection; ^a^–none include who didn’t drink alcohol in their lifetime or in the last 12 months

* = P < 0.05

** = P < 0.01 and

*** = P ≤ 0.001.

## Discussion

Risky sexual behavior is a major public health problem globally. Similarly, in Ethiopia, the magnitude of risky sexual behaviors was increasing alarmingly and puts adolescents and youth at higher risk for negative health outcomes [16]. Therefore, assessing the magnitude of risky sexual behavior and its determinants both at the individual and community level will make a significant contribution to the design of effective public health intervention programs.

The finding of this study revealed that 25.5% of unmarried men had a sexual debut before the age of 18 years. This finding is lower than found among a sample of unmarried most-at-risk young people in Cambodia [24] but it is higher than a study conducted in Nigeria [25]. This high magnitude of early sexual initiation, in Ethiopia, suggests that unmarried sexually active men may need special attention to reduce the burden of negative health outcomes related to early sexual initiation. Therefore, awareness creation on the negative effect of early sexual initiation for young through the use of mass media, school teachers, and parents might be an essential public health intervention in Ethiopia.

The current study showed that 3.4% of unmarried men had multiple sexual partners which was lower than a study conducted among university students in Ethiopia, which reported (18.6%) [26]. In this study, 15.1% of the respondents reported inconsistent condom use with their most recent sexual partner. The finding is much lower than a study done in India [27]. It could be attributed to an unfavorable attitude toward using a condom or the social norms in which the youth live and grow. For instance, using a condom is considered as distrusting sexual partners by some people in Ethiopia [28]. This finding highlights the need for continuous health education and behavioral change on consistent condom use among sexually active unmarried men to reduce the incidence of sexually transmitted infections including HIV.

Similar to the previous studies [29, 30], the current finding revealed that unmarried men who have completed primary and secondary levels of education had lower odds of risky sexual behavior. This could be better explained due to the fact that educated men might be aware of the consequence of risky sexual behavior and the effect of STIs including HIV [31]. In addition, educated men might have access to information related to the consequence of risky behavior. The finding of this study implies that education can play a crucial role in informing society about the consequence of risky behavior. However, this study finding contradicts the previous study conducted in developing countries that reported males with higher educational attainment were more likely to engage in higher-risk sexual behavior [32].

Previous studies revealed that employment is associated with a reduced risk of engaging in risky sexual behavior [30, 33]. However, in the current study, we found that unmarried men who had been working within the 12 months preceding the survey had higher odds of having risky sexual behavior as compared to unemployed men. This might be because men who were working can have a good income which might predispose them to have had more than one concurrent sexual partner [34].

The finding of this study revealed that men in the middle wealth index quantile had a lower odds of having risky sexual behavior as compared to men in the lower wealth index quantile. Previous studies [17, 34] showed that individuals who had high income might have the opportunity to practice what they desire which in turn increases the probability of risky sexual behavior. However, this is in contrast with the findings of a recent study done in Sub-Saharan Africa [35]. This finding suggests that the high wealth index quantile may be the root cause of risky sexual behavior among this segment of the population in Ethiopia.

In this study, men who had ever been tested for HIV had a higher odds of risky sexual behavior. This finding is supported by a study done in Senegal [36] revealed that prior HIV testing increases the risk of practicing risky sexual behavior especially inconsistent condom use with their regular partners, especially in individuals who tested HIV negative. This might be associated with the psychological effects of receiving a negative test. Receiving a negative test, especially after a risk exposure, may be understood as permission for risk, reinforcement that risk does not have harmful consequences. On the contrary, several studies revealed that prior HIV testing positive impact on reducing risky sexual behavior [37, 38]. Past evidence showed that the benefit of HIV testing in reducing risky sexual behavior is different among individuals who tested positive and those who tested negative [39]. Among individuals who tested positive, HIV testing makes people aware of their HIV status and promotes safer sex behaviors [39]. On the contrary, testing negative for HIV may increase risky sexual behaviors among sexual partners especially inconsistent condom use [40].

In this study, men who drunk alcohol almost every day in the last 12 months prior to the survey had a higher risk of practicing risky sexual behavior as compared to non-drinkers. This association might be explained by the alcohol myopia model [41] which states that alcohol reduces cognitive capacity and causes people to focus only on the cues that are most significant in the environment like the immediate pleasure of sexual contact. Less salient cues like condom use and suspicion that the sexual partner could be HIV infected are less likely to be acted upon by an intoxicated person.

The Internet has its advantages in terms of providing necessary information on reproductive health and healthy sexual relationships, but many studies have shown that inappropriate Internet usage negatively influences youth sexual behaviors. Studies done in different parts of the world showed that the majority of young individuals with internet access fell on pornographic sites, which influenced their sexual behavior such as engaging in oral sex, having multiple sexual partners, early sexual initiation, and homosexuality [42–44]. Similarly, in the current study, we found that using the Internet almost every day is a precipitating factor for risky sexual behavior which is consistent with previous studies [42, 43] which have shown that individuals who spend more time online are more likely to watch sexual content such as pornography which may encourage the early onset of sexual intercourse. Using the Internet almost every day may have a major impact on men’s behavior through imitation and copying of risky sexual acts found on the Internet and they may be more motivated to practice what is observed online. Besides, the sexually explicit information found on the Internet might be inaccurate and harmful which encourages sexual acts without any emotional connection.

In the current study, we found a negative association between a higher proportion of media exposure and risky sexual behavior. Men residing in communities with a high proportion of media exposure had a lower odds of having risky sexual behavior as compared to men residing in communities with a low proportion of media exposure. This might be due to the fact that mass media play a vital role in increasing awareness about the consequences of risky sexual behavior. This ultimately helps in the change in sexual behavior of men like consistent condom usage. The result of this study could indicate the importance of media exposure at the community level to reduce risky sexual behavior. Therefore, the Ethiopian government should increase the level of community-level media exposure through different media like radio, television, newspaper, leaflet, and poster.

In this study, the region was another community-level determinant of risky sexual behavior. Men from Tigray, Amhara, Oromia, Somalia, SNNP, and Harari regional states had a lower odds of having risky sexual behavior as compared to men from Addis Ababa. The possible explanation for this might be due to the fact that men living in large cities like Addis Ababa may have access to sexually explicit materials which might influence men’s sexual behavior. The other possible explanation might be due to regional variation in socio-cultural practices in Ethiopia. Hence, special focus should be given for sexually active men residing in large cities while designing programs and different interventions.

The main strength of this study was the use of models that accounted for clustering in the data, which if ignored underestimates variance while overestimating significance. However, the study has also several limitations. First, the cross-sectional nature of the analysis does not support causality between independent and dependent variables. Second, the outcome variable has relied on men’s self-reported sexual behavior which may be subject to social desirability and recall bias.

## Conclusion

In general, this study has revealed that a significant proportion of sexually active unmarried men in Ethiopia have practiced risky sexual behavior. This significantly results in an increase in the incidence of HIV/AIDS, unwanted pregnancies, and unsafe abortions in the country. Individual-level factors, like currently working, history of HIV testing, drinking alcohol almost every day, and using the Internet frequently are risk factors that increase the odds of risky sexual behavior. To reduce the incidence of HIV, an intervention should also be designed against the community-level factors found to increase the odds of risky sexual behavior. The Ethiopian government particularly the Ministry of Health should design a policy to improve the community-level media exposure through different media like radio, television, newspaper, leaflet, and poster.

## List of abbreviations

AOR: Adjusted Odds Ratio
COR: Crude Odds Ratio
EDHS: Ethiopia Demographic Health Survey
HIV: Human Immune-Deficiency Virus
